# The Role of the CREB Protein Family Members and the Related Transcription Factors in Radioresistance Mechanisms

**DOI:** 10.3390/life11121437

**Published:** 2021-12-20

**Authors:** Gianmarco Stati, Francesca Passaretta, Florelle Gindraux, Lucia Centurione, Roberta Di Pietro

**Affiliations:** 1 Department of Medicine and Ageing Sciences, G. d’Annunzio University of Chieti-Pescara, 66100 Chieti, Italy; francesca.passaretta@gmail.com (F.P.); l.centurione@unich.it (L.C.); r.dipietro@unich.it (R.D.P.); 2 Laboratoire de Nanomédecine, Imagerie, Thérapeutique EA 4662, Université Bourgogne Franche-Comté, 25030 Besançon, France; fgindraux@chu-besancon.fr; 3 Service de Chirurgie Orthopédique, Traumatologique et Plastique, CHU, 25030 Besançon, France

**Keywords:** CREB/ATF, NF-κB, radioresistance, ionising radiation, galactic cosmic rays, radiotherapy, human cancer, space flight

## Abstract

In the framework of space flight, the risk of radiation carcinogenesis is considered a “red” risk due to the high likelihood of occurrence as well as the high potential impact on the quality of life in terms of disease-free survival after space missions. The cyclic AMP response element-binding protein (CREB) is overexpressed both in haematological malignancies and solid tumours and its expression and function are modulated following irradiation. The CREB protein is a transcription factor and member of the CREB/activating transcription factor (ATF) family. As such, it has an essential role in a wide range of cell processes, including cell survival, proliferation, and differentiation. Among the CREB-related nuclear transcription factors, NF-κB and p53 have a relevant role in cell response to ionising radiation. Their expression and function can decide the fate of the cell by choosing between death or survival. The aim of this review was to define the role of the CREB/ATF family members and the related transcription factors in the response to ionising radiation of human haematological malignancies and solid tumours.

## 1. Introduction

Outside of the Earth’s protective magnetosphere, crews are exposed to galactic cosmic rays (GCR) and solar proton events (SPE) that occur when particles emitted by the sun, mostly protons, become accelerated in the interplanetary space due to a coronal mass ejection shock [1]. The exposure to GCR in the form of high-energy (HZE) ions, secondary protons, and neutrons has high linear energy transfer values that evoke complex DNA and other cellular damage [2]. In the purpose of controlling the health risks associated with the unique hazards of space flight, highly sophisticated systems have been developed [3]. A key question that impacts risk assessment is how cancers caused by HZE radiation compare to either radiogenic cancer induced by ground-based radiation [4]. As a unifying concept, NASA studies have sought to examine how space radiation exposure induces genetic and epigenetic modifications, noted as the hallmarks of cancer onset [5]. Due to the lack of human epidemiological data related to the types of radiation found in space, the current research utilises a translational approach which includes advanced human cell-based model systems exposed to space radiation simulants connected with human molecular pathways [6]. This approach allows relating the biological effects of space radiation to effects from similar exposure to ground-based gamma rays and X-rays to extrapolate the results to large human epidemiological cohorts [6,7]. Among the molecules involved at the cellular level, the CREB/ATF family plays a crucial role.

The CREB protein was initially described as a cAMP-responsive transcription factor involved in the regulation of the somatostatin gene [8]. Today, it is known to modulate gene transcription through binding to DNA sequences known as the cAMP response elements (CREs) [9]. In the human genome, there are approximately 750,000 of these CREs, although most of them are not available for protein binding as their cytosine methylation physically prevents these interactions [10]. The CREB/ATF transcription factors have key roles in cell survival, proliferation, and differentiation, as well as in apoptosis and adaptive responses [11]. Nuclear factor-κB (NF-κB) appears to be the most important CREB-related transcription factor [12]. Increased NF-κB activity can be considered as a hallmark of different diseases, such as human leukaemia, lymphoma, and other types of cancers [13]. NF-κB activity can be induced by ionising radiation, which appears to promote enhanced survival of human leukaemia K562 cells [14]. Indeed, a link has been shown between constitutive NF-κB activity, basal apoptosis, and radiosensitivity in breast carcinoma cell lines [15]. Furthermore, high NF-κB activity in human cancers can promote apoptosis suppression and radiotherapy resistance [16]. 

The present review is focused on the complex biochemical interactions of the CREB/ATF family members and the related transcription factors in the response to ionising radiation of human haematological malignancies and solid tumours.

## 2. Ionising Radiation: Radioresistance and Radiosensitivity

When atoms disintegrate, energy is released in the form of ionising radiation, which includes electromagnetic waves (e.g., gamma rays, X-rays) and particles (neutrons, alpha and beta particles). They can have a sufficient level of energy to result in ionisation of atoms and molecules [17]. When an organism is exposed to ionising radiation, the energy is absorbed by the atoms of the biomolecules in the cells, which become ionised or excited [18]. This can result in the formation of free radicals and reactive oxygen species (ROS) [19]. This build-up of ROS can then lead to several types of cellular damage in the form of genetic effects with direct alterations of DNA integrity, epigenetic effects with modifications of the DNA, or what are known as bystander effects [20], as further defined below. 

**Figure 1:**
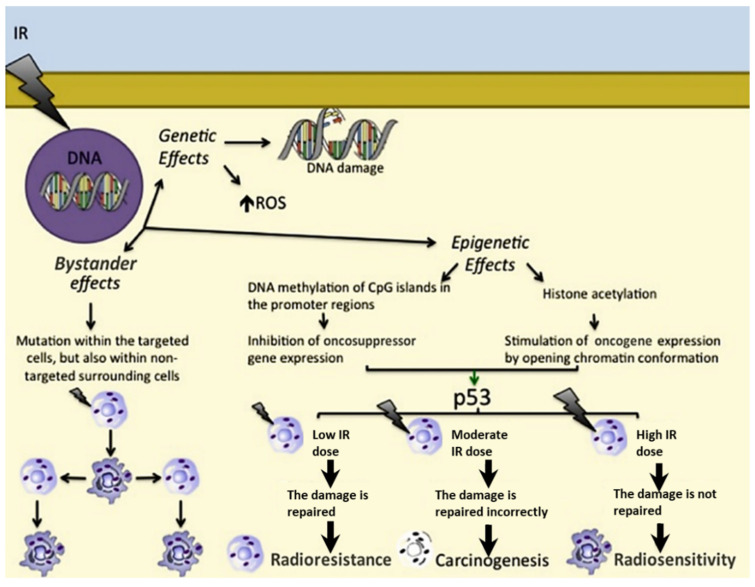
Genetic effects, epigenetic effects, and bystander effects of ionising radiations. The role of p53 depending on the IR dose is as follows: in response to a low IR dose, p53 induces cell survival and radioresistance; upon a moderate IR dose, p53 is not able to correctly repair damaged DNA, leading to carcinogenesis; upon a high IR dose, p53 is not able to repair irretrievably damaged DNA, resulting in radiosensitivity. **→** = activation; **→** = consequentiality; **↑** = overexpression.

To better understand the distinction between targeted and nontargeted effects of ionising radiation, it is necessary to consider the biological responses not just of the irradiated cells, which will mainly be killed, but particularly of the surviving cells in the close vicinity that have not undergone direct irradiation. Genomic instability is seen as the appearance of genetic abnormalities in the “offspring” of both the surviving irradiated cells and their close neighbours. They may exhibit adaptive responses, which are characterised by changes in their radiation susceptibility—in a protective manner—following the initial dose of radiation (i.e., the challenge dose). This may also apply to the cells previously exposed to one or more low doses of radiation [14] since it was seen that exposure to any radiation dose is pivotal in terms of risks [15]. Indeed, carcinogenesis is characterised by a multistage process where different target genes undergo modifications of their expression after radiation exposure in a dose-dependent manner. DNA repair genes, proto-oncogenes, and tumour suppressor genes shall be responsible for the deregulation of the cell cycle and the alteration of the apoptotic pathway [16,17]. In addition, the expression of small non-coding RNAs is affected by ionising radiation [18]. As indicated above, the main cellular effects of ionising radiation can be divided into three groups: genetic effects, epigenetic effects, and bystander effects (Figure 1). 

### 2.1. Genetic Effects

The direct genetic effects arising from radiation-induced DNA lesions are single-strand breaks or double-strand breaks, which lead, respectively, to the activation of the DNA repair process and apoptosis [21,22]. Furthermore, accumulation of ROS can also lead to indirect effects of ionising radiation that can cause structural and functional defects of the cellular nucleic acids, proteins, and lipids. This intracellular damage accumulation can lead to tumour development due to the increased DNA mutation rate [23]. In other cases, ROS-mediated oxidation can result in chromosomal rearrangements and errors in recombination processes [21,22] or damage to mitochondrial DNA. All these events have important roles in the development of secondary malignancies after radiotherapy [24].

### 2.2. Epigenetic Effects

Low ionising radiation doses can induce epigenetic effects at the cellular level through the involvement of ROS, and they can also promote carcinogenesis [25]. The main epigenetic modifications are DNA methylation of CpG islands of the gene promoter regions, with inhibition of gene expression [26] and histone acetylation with gene overexpression [27,28]. 

### 2.3. Bystander Effects

The signals that arise from radiation-induced bystander effects can be transmitted through tissues by means of direct intercellular contacts, similarly to local paracrine effects, or by means of the more remote actions of molecules, similarly to endocrine effects [29]. Ionising radiation can thus induce mutations not only within the nontargeted surrounding cells, but also in distant tissues [30]. This can be due to communication between cells through their gap junctions [30], various ligands and their receptors [31], and other released factors (e.g., ROS, interleukin (IL) 1, and transforming growth factor-β1) and their receptors [32,33]. Indeed, carcinogenesis induction has been proved for nearby unirradiated cells [34].

Overall, cell response to ionising radiation depends on the balance between cell cycle progression, DNA repair, and apoptosis [35]. P53 plays a key role here, as the cell cycle arrest induced in response to DNA damage can allow DNA repair or induce apoptosis [36]. This depends further on the dose of the ionising radiation, whereby low doses can allow DNA repair, which induces cell protection (i.e., radioresistance), while high doses trigger apoptosis, and hence cell death (radiosensitivity) [37]. 

Radiation-induced bystander effects appear to be correlated to tumour induction through enhanced radioresistance or increased cell proliferation [38]. Here, nitric oxide (NO) has a crucial role as it is produced and released by irradiated cells and it is able to act on nonirradiated bystander cells [39]. Moreover, the NO effects depend on the radiation dose as while NO can be cytotoxic and thus cause DNA damage leading to cell death, it can also be cytoprotective by protecting cells from apoptosis. The pro- or antitumoral properties of NO depend on local concentration, exposure time, redox state of the cell and its compartmentalization [38,40]. 

Thus, cells can respond to a low or priming dose of radiation by acquiring radioresistance as defined by the so-called radiation-induced adaptive response [41]. Based on this evidence, bystander effects can make cells radioresistant [42]. This appears to be caused by stimulation of the genes involved in DNA repair [43] and suppression of p53-dependent responses [44].

## 3. CREB Family Members and CREB-Binding Proteins

CREB is a basic region/ leucine zipper motif (bZIP) transcription factor as it contains a leucine zipper domain that facilitates dimerization and mediates its DNA binding [45,46]. The CREs to which these CREB dimers bind have the sequence TGACGTCA [47]. The phosphorylation of CREB at Ser133 promotes its binding to the coactivator CREB-binding protein (CBP), which facilitates transcription [48] as this binding promotes chromatin loosening through histone acetylation [49]. This pathway is involved in several biochemical metabolic cell processes, including glucose homeostasis, cell survival, proliferation, differentiation, apoptosis, memory, and learning [50]. The CREB/ATF transcription factors include three genes that are homologous: *CREB* itself, cAMP response element modulator (*CREM*), and *ATF-1* [51]. Their gene products are thus three highly homologous proteins: CREB, CREM, and ATF-1. The CREB/ATF family to date includes CREB-1, CREB-2 (recently named ATF-4), CREB-3, CREB-5, CREM, ATF-1 (also known as TREB36), ATF-2 (also known as CRE-BP1), ATF-3, ATF-5 (also known as ATFX), ATF-6, ATF-7, and B-ATF [52]. As indicated above, they have bZIP domains, the sequences of which define their formation of homodimers or heterodimers. These heterodimers can include not just those with other CREB/ATF members, but also those with other bZIP-containing proteins, such as activator protein-1 (AP-1), C/EBP, and the Fos, Jun, and Maf proteins [53].

CREB, CREM, and ATF-1 have been characterised, and they are known to regulate gene transcription through their binding to CREs. In contrast, the properties of the structurally and functionally more distant ATF-2, ATF-3, and ATF-4 are not yet fully understood, although they are stress response proteins [54,55]. ATF-3 has been indicated as an adaptive response gene activated by several cell signals, which include those of the cytokines and genotoxic agents, and physiological stress [56]. ATF-3 has also been implicated in host defence systems against invading pathogens and cancer development [57,58]. ATF-3 expression is ubiquitous in all tissues, and it is localised in the nucleus; in the absence of cell stress, ATF-3 levels remain low [56]. However, ATF-3 is rapidly induced under cell stress, including during hypoxia, DNA damage, and heat or cold shock [56]. In contrast, in neuronal cells, ATF-4 has been shown to migrate from the cytoplasmic leaflet into the nucleus following activation of γ-aminobutyric acid receptors. ATF-4 has thus been suggested to be involved in neuronal plasticity, whereby it appears to couple receptor activity to gene expression [59].

Human CBP and its paralogue p300 are generally functionally interchangeable, although they each also have their own unique functions [60]. They have multiple domains for binding to over 40 transcription factors, and they are multifunctional adaptor proteins that have acetyl transferase activities for transcription factors and histones [61]. They are also important for cell cycle progression and cell differentiation where they can interact with components of the RNA polymerase II holoenzyme, with transcription factors, nuclear hormone receptors and their coactivators [62]. They have been shown to support normal cell differentiation and cell cycle arrest through interaction with GATA-1 (transcription activator/repressor); however, they can also promote cell cycle progression through interactions with c-Myb (transcription activator) and PU.1 (Ets family transcription factor). Several mutations have been described for the transcription factor-binding domains of these genes across different tumours [63]. 

Following identification of the molecular mechanisms through which CREB acts as an inducible regulator of such transcription factors, CREB has become the focus of many investigations, particularly with its involvement in various signalling pathways under both normal and pathological conditions. Furthermore, CREB interacts with other nuclear factors through its transactivation domain, which includes the Q2 domain, a constitutively active glutamine-rich domain, and the kinase-inducible domain that is regulated by cellular kinases. The interaction of the Q2 domain with TATA-binding protein-associated factors promotes constitutive activation, while the kinase-inducible domain promotes isomerisation through recruitment of coactivators CBP and p300 to gene promoters, with activation seen only following phosphorylation of Ser133 by specific cellular kinases [64].

## 4. NF-κB and p53: Two Crucial CREB-Related Transcription Factors

Most tumour cells show high levels of constitutive NF-κB activity, with increased cell survival promoted through antagonism of apoptotic pathways [65]. A key role of the NF-κB pathway is the promotion of the innate immune response through transcription of proinflammatory mediators, including chemokines, cytokines, antimicrobial peptides, and adhesion molecules [66,67]. The NF-κB family members include NF-κB1 (p50, p105), NF-κB2 (p52, p100), RelA (p65), c-Rel, and RelB [68]. Activation of NF-κB can occur through phosphorylation and proteolysis of the inhibitor of κB (IκB) or through an IκB-independent pathway [69] and as a stress-sensitive transcription factor. In fact, NF-κB is involved in regulation of the expression of stress-responsive genes [70]. In the nucleus, the interaction between the NF-κB family member RelA and CBP or p300 is required to promote NF-κB-dependent transcription of genes that induce proinflammatory responses. The RelA subunit activity involved in this interaction is potentiated by CBP and p300 [71]. P300 regulates cell sensitivity and has a proapoptotic function following cell damage [72]. The correlation between NF-κB and phosphorylated CREB is due to their interaction with CBP/p300 in the same region [73]. Consequently, this competition would lead to CREB-dependent inhibition of NF-κB activity [74]. In addition, CREB can induce an NF-κB-dependent antiapoptotic response in macrophages, which promotes macrophage survival and thus enhances the immune response [75]. 

Substantial evidence points to a critical role of the p300/CBP coactivators in p53 responses to DNA damage [76]. Interestingly, p53 was the prototype to demonstrate that p300, CBP, and the associated P/CAF acetylase can acetylate nonhistone transcription factors [77]. Both p300 and CBP can interact with p53, but only p300 appears to be involved in p53 stabilisation after DNA damage and p53-dependent apoptosis. The tumour suppressor protein p53 has key roles in prevention of the development of cancers, and it is inactivated in many human malignancies [78]. Indeed, p53 regulates expression of the genes involved in cell cycle control and apoptosis induction [79] and at the same time can induce transcription of DNA repair enzymes to promote cell survival [80]. It can be stimulated by cell stresses, such as oxidative stress, hypoxia, ionising radiation, and carcinogens [81], and its levels rise after DNA damage, whereas they are low in both normal and neoplastic cells under normal physiological conditions [82]. The uncovering that the *TP53* gene physiologically expresses, in a tissue-dependent manner, several p53 splice variants (isoforms) provides an explanation for its pleiotropic biological activities [83]. In fact, among the different isoforms of p53, some have oncogenic potential, while others show oncosuppressor properties or, in some cases, can have both functions, depending on the cellular context [22]. The p53 family of proteins also includes p63 and p73 that can induce apoptosis by mediating cell cycle arrest at G2/M and G1/S [84]. Importantly, *TP53* is deactivated in most of the human solid tumours due to missense mutations and deletions that impair its transcriptional function [85]. In cancer, the presence of p53 mutants leads to gain-of-function phenotypes due to increased cell growth and cell motility, coupled with carcinogenesis and chemoresistance [86]. The gain-of-function phenotype arises through chromatin changes following histone acetylation, which results in interactions between the STAT1/STAT2 complex and CBP/p300 on the NF-κB2 promoter [87]. 

## 5. CREB Family Members: An Active Role in Tumours

**Figure 2:**
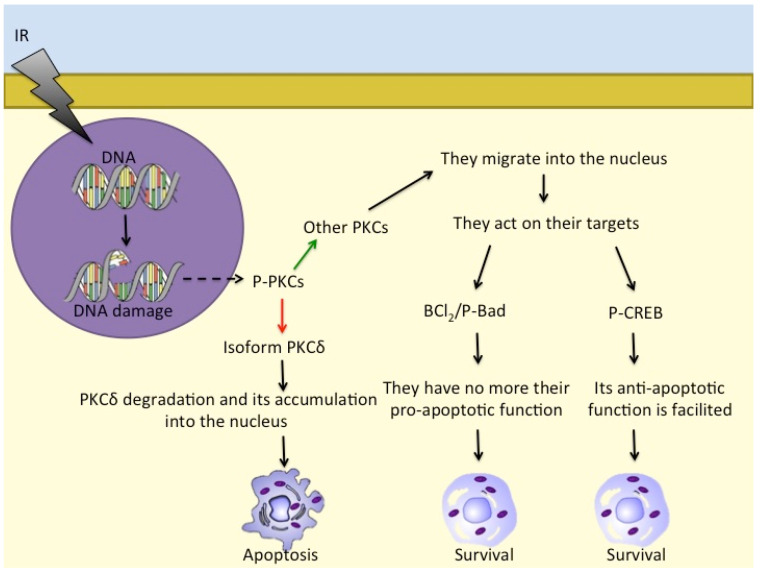
Different PKC isoforms respond to DNA damage in different ways: while PKCδ accumulates in the nucleus and induces apoptosis, other PKCs act on Bcl2/P-Bad and on P-CREB to promote cell survival. - - -→ = response to DNA damage; ––> = activation; ––> = inhibition; ––> = consequentiality.

Several recent studies are proposing CREB/ATF and the related nuclear transcription factors as potential prognostic biomarkers [88]. It is well-known that CREB expression mediated by phosphorylation is essential for major cell survival functions [88]. Tumour cells have developed various mechanisms to achieve constitutive activation of CREB, including gene amplification, chromosome translocation, and inactivation of tumour suppressor genes, leading to uncontrolled proliferation of cells [89]. Therefore, it is hypothesised that CREB is directly involved in the pathogenesis of a variety of cancers (Table 1), including hematological malignancies [90]. CREB is often overexpressed in hematopoietic tumours [88,89,91], and its implication in the radiation response of lymphoid neoplastic cell lines has been demonstrated [92,93]. Overexpression of CREB is known to be associated with increased cell proliferation and migration and reduced apoptosis, processes that are both directly and indirectly linked to malignant transformation of cells [94]. In human melanoma, CREB and ATF-1 have been reported to act as survival factors and favour tumour growth and metastasis [95,96]. During the progression of melanoma, CREB and ATF-1 show changes in their expression, along with the loss of the AP-2α transcription factor: indeed, CREB expression correlates directly with metastatic transformation [96], whereas ATF-1, which is absent in normal melanocytes, is then expressed in the metastatic melanoma cells [97]. In different cell types, CREB and ATF-1 have been shown to act as negative regulators of apoptosis as these proteins appear to rescue cells from apoptotic death through upregulation of Bcl-2 expression, with binding of CREB and ATF-2 to a CRE domain within the Bcl-2 promoter (Figure 2).

CREB also has an important role in breast cancer through its interactions with tumour-promoting G-protein-coupled receptor 81 (GPR81), which is involved in the promotion of angiogenesis and breast cancer cell survival in the tumour microenvironment [98]. For instance, in the MCF7 breast cancer cell line, GPR81 activation has been shown to promote CREB phosphorylation, and thus the nuclear translocation of the active form of CREB. PI3K/Akt suppression was shown to block this GPR81-induced activation of CREB and subsequently inhibit expression of the proangiogenic mediator amphiregulin (AREG) and thus angiogenesis. Therefore, the pathway that involves PI3K/Akt and CREB can promote tumour-associated angiogenesis [98]. 

High constitutive expression of CREB has been observed in the immortalised Jurkat T lymphocyte cell line [99]; therefore, CREB might sustain the proliferation of lymphoid leukaemia cells. Indeed, the involvement of CREB in the pathogenesis of myeloid and lymphoid leukaemia has already been demonstrated [100]. Interestingly, a recent study revealed the association between CREB1 overexpression with metastasis, tumor stage, and poor outcomes in gastric cancer [101]. Moreover, CREB has an important role in the progression of hepatocellular carcinoma (HCC) by promoting angiogenesis and resistance to apoptosis [102]. It is possible that CREB confers drug resistance to HepG2 cells through activation of the human multidrug resistance 1 (*mdr1*) gene and consequent increase in P-glycoprotein (P-gp) [103], along with activation of the *bcl-2* antiapoptotic gene [104]. In neuroendocrine prostate cancer, the CREB/G-protein-coupled receptor kinase (GRK) 3 axis promotes neuroendocrine differentiation of the prostate cancer cells, whereas CREB activation is mediated by GRK3. Hence, it appears that GRK3 represents a drug target for the treatment of patients with aggressive prostate cancers [105]. Moreover, GRK3 has oncogenic roles in different human cancers including prostate cancer [106], acute myeloid leukaemia [107], and pancreatic cancer [108], and its activity can be regulated by CREB in lung cancer [109]. Thus, in these human cancers, overexpression, or increased activity of CREB promotes disease progression [100]. 

## 6. CREB Family Members and Related Transcription Factors in Radiotherapy of Solid Tumours and Leukaemia

### 6.1. Cell Responses to Ionising Radiation 

Ionising radiation is responsible for the loss of cell proliferation and for cell death by apoptosis or necrosis. Of note, there are two types of apoptosis: fast apoptosis, which occurs during the interphase, before cell division, and after the G2 block that is induced by radiation; and late apoptosis, which occurs after one or more cell divisions [110]. Some cells, such as lymphocytes, thymocytes, and intestinal crypt cells, are particularly radiosensitive, and when they are irradiated, they undergo fast apoptosis; when mouse leukaemia cells are irradiated, they experience G2 block, and mainly undergo apoptotic death [111].

As a response to genomic stress, activation of p53 can result in cell cycle arrest or cell death by apoptosis, and this can also contribute to DNA repair processes [112]. The mouse double minute (Mdm) 2 protein is a crucial regulator of p53. In mice, inactivation of the *mdm2* gene shows early embryonal lethality [113]. Mdm2 has a dual relationship with p53. When Mdm2 binds to p53, this can inhibit the transcriptional function of p53, which also results in complete elimination of p53 by proteolytic degradation. At the same time, p53 can bind the *mdm2* gene, which stimulates its transcription. Therefore, this defines a negative feedback loop that appears to serve to rapidly terminate the p53 response after effectively dealing with the p53-activating stress signal [114]. Various mechanisms have been proposed to explain the p53 fluctuations that are observed in cell populations [115]. However, considering the continuous effects on cells of acute ionising radiation, the complex cell responses that can be activated to fight against DNA damage still need to be addressed further at the level of the single cell. For oncogenes and toxins that have p53 regulatory functions, their degradation kinetics can be used to quantitatively predict outcomes of the cell responses to DNA damage induced by different doses of ionising radiation [116]. The retinoblastoma protein (Rb) and CBP/p300 have more complex influences on the p53/MDM2 interactions. Here, the binding of Rb to MDM2 prevents the MDM2 destabilisation of p53 while the Rb/MDM2 complex continues to bind to p53 and inhibits the transactivation mediated by p53 [117]. Thus, this indicates how the p53 inhibition and destabilisation functions of MDM2 can be individually deciphered. Regarding CBP/p300, these coactivators of p53 are also required for p53 degradation mediated by MDM2. When MDM2 lacks its p300-binding domain, it can no longer destabilise p53, although the MDM2 and p53 binding appears not to be affected [118].

There are further proteins that are involved in cell responses to ionising radiation, such as PKC family members [92,119]. PKCs are serine/threonine kinases and comprise (at least) 12 different isozymes. PKCδ releases a 40 kDa fragment by proteolysis when cells are exposed to ionising radiation and also to DNA-damaging drugs that leads to apoptosis [120]. Furthermore, in response to irradiation, activated PKCs regulate P-Bad, Bcl-2, and CREB to prevent apoptosis and induce pro-survival signalling [121]. PKCs also translocate into the nucleus to phosphorylate its targets [122,123] (Figure 2). 

### 6.2. CREB and Other Factors in Radioresistance and Radiosensitivity

Radiotherapy is designed to induce DNA double-strand breaks, which would then lead to elimination of cancer cells via apoptosis [124]. However, the efficacy of radiotherapy treatment against cancers also depends on the toxic side effects, which can impede dose escalation. Furthermore, as indicated above, cancer cells might instead develop radioresistance through mechanisms related to DNA repair responses. When cells are exposed to ionising radiation, there is activation of transcription factors like AP-1 and NF-κB [125]. The consequent induction of specific genes and synthesis of their protein products might then provide the cells with radiation resistance. When the constitutive levels of NF-κB are high, cells also show relatively high resistance to radiation therapy [126]. The Daudi and Ramos cell lines (human Burkitt lymphoma) show sensitivity to relatively low radiation doses (i.e., 1–5 Gy), with reduced cell viability due to necrosis and apoptosis, and the cell cycle blocked in the G_2_/M phase [93]. The less radiosensitive Ramos cells show expression of a mutated form of p53 and a constitutively activated NF-κB pathway. These cells are sensitive to an ionising radiation dose of 3 Gy, where they show an early increase in the expression of CREB and a dose-dependent upregulation of expression of NF-κB [93]. Interestingly, increased cellular levels of CREB have proapoptotic effects, while NF-κB upregulation can be linked to necrosis, at least in vitro and after 3 Gy ionising radiation [93]. 

Concerning the regulation of NF-κB, normally, its nuclear translocation and activation are prevented by the “super-repressor” IκB, but stimuli such as TNFα and ionising radiation can degrade, and thus remove, IκB, leaving NF-κB free to translocate into the nucleus and activate its target genes [127]. Therefore, as NF-κB is activated in several types of cancer, this might provide the cells with intrinsic radioresistance or promote radioresistance. Indeed, NF-κB activity induced by ionising radiation appears to enhance the survival of K562 human leukaemia cells [14]. Furthermore, in breast carcinoma cell lines, a link has been reported for constitutive NF-κB activity, basal apoptosis, and radiosensitivity [128]. This indicates that the higher levels of NF-κB seen for human tumours can both suppress apoptosis and promote radioresistance. Inhibition of the NF-κB expression generally increases the apoptotic response when cells are under radiation therapy [129], and as indicated above, NF-κB expression is upregulated in certain tumour cells in response to radiation, and to chemotherapeutic drugs [130]. Instead, K562 leukaemia cells have shown a different strategy for resistance to apoptosis induced by ionising radiation that is modulated through protein kinase C (PKC) δ and NF-κB [14]. 

CREB has an active role in prostate cancer, where radiation therapy is the first-line treatment [131]. In the human prostate, neuroendocrine cells represent one of three types of epithelial cells [132]. These neuroendocrine cells can promote growth of the surrounding tumour cells through their release of neuropeptides [133]. Along with CREB, a role for ATF2 has been indicated in prostate cancer. Indeed, it has been hypothesised that ATF2 acts as a shuttling protein as it moves between the cytoplasm and the nucleus [134]. Ionising radiation can induce reversible differentiation of neuroendocrine cells, which leads to the loss of their neuroendocrine properties. Here, CREB and ATF2 might have opposing effects as it appears that accumulation of ATF2 in the nucleus antagonises the signalling pathway involved in the phosphorylation of CREB that is induced by ionising radiation. After exposure to ionising radiation, the differentiated cells show increased proliferation, thus losing their neuroendocrine-like properties [135]. In this case, radiotherapy also gives tumour cells the possibility to survive the treatment and contribute to tumour recurrence.

Radioprotection of human leukaemia cell lines can also be linked to the stability of peroxiredoxins (Prx) [136]. They are a very large and conserved family of small peroxidases that conserve the thioredoxin-dependent catalytic activity that protects cells from oxidative damage produced by H_2_O_2_, organic hydroperoxides, and peroxynitrite [137]. The effects of ionising radiation in leukemia cells are of oncologic interest since high doses of whole-body gamma radiation can be employed before bone marrow transplantation. However, PrxII expression levels have been correlated with radioresistance or administration of certain anticancer drugs in radioresistant solid tumours, including breast cancer, glioblastoma, and head and neck cancer, as well as in tissue isolated from head and neck patients who do not respond to radiation therapy [138].

### 6.3. CREB and Related Transcription Factors as Possible Targets in the Treatment of Tumours

CREB appears to be a therapeutic target for cancer treatment due to its role in the development, maintenance, and progression of tumours [94]. This was also suggested by the downregulation of the inducible cyclic AMP early repressor (ICER) in bone marrow cells from patients with acute myeloid leukaemia, where altered CREB expression was observed. There are several different ways in which the CREB function might be inhibited in tumour cells [94]. First, a dominant-negative CREB mutant, known as KCREB, can be used to inhibit transcription of CREB, which arises through the formation of heterodimers of KCREB with wild-type CREB. In metastatic tumour cells in vitro and in vivo, KCREB has been shown to reduce the metastatic potential [95]. Secondly, CRE “decoy” oligonucleotides might inhibit *CREB* gene transcription and tumour growth [139]. Thirdly, silencing of CREB expression might reduce anchorage-independent growth of tumour cells, along with cell cycle arrest. This would lead to apoptosis accompanied by enhanced tumour immunogenicity [140]. Finally, many kinase inhibitors can be used to inhibit the CREB and CBP interactions with their CREs [141]. 

Pharmacological inhibition of IKK-NF-κB might also represent an interesting approach to potentiate the apoptotic effects of irradiation. Indeed, a suppressor of the IKK complex was reported to make lung cancer cell lines more apoptosis-susceptible following their irradiation [142]. Furthermore, curcumin, which interferes with activation of the inhibitor of NF-κB kinase (IKK), showed greater radiation-induced apoptosis against PC-3 prostate cancer cells [143]. Instead of targeting NF-κB itself, the effector genes of NF-κB might represent better drug targets for the enhancement of radiosensitivity on the basis that in tumour cells that are radioresistant, activation of these genes might be predominant. In an investigation of genes that are upregulated in human keratinocytes following long-term irradiation, Chen et al. (2002) [112] reported that six genes showed upregulation as putative targets for NF-κB. By using a mutant IκB, three of these genes, cyclin B1, cyclin D1, and human inhibitor of apoptosis protein (HIAP) 1, were downregulated, while the other three upregulated genes, Bcl-2-associated athanogene (BAG) 1, thyroid transcription factor (TTF), and fibronectin, were not downregulated. The genes that were downregulated following NF-κB inhibition are associated with significantly decreased cell survival, and thus they might have crucial roles in radioresistance. 

For hepatocyte malignancy in HCC, radiotherapy is the most common treatment choice [144]. Unfortunately, as both cancer and healthy cells are killed, this treatment comes with multiple side effects for the cells. However, as Fuchs-Tarlovsky (2013) [145] reported, administration of antioxidant nutrients prior to or combined with radiation therapy protected nontumour cells against the free radicals generated to kill the tumour cells during the irradiation.

P53 can also be considered as a target in anticancer therapies that use different small molecules that have been shown to restore the function of wild type p53. One of these is the cis-imidazoline analogue nutlin-3 which prevents p53 degradation and was shown to induce apoptosis in p53-deficient colorectal carcinoma cells and in an HCC cell line through p73 activation [146,147]. Previous preclinical studies showed the therapeutic use of nutlin-3 for haematological malignancies, including acute myeloid leukaemia [148], acute lymphoblastic leukaemia [149], and B cell chronic lymphocytic leukaemia [150]. In a human head and neck cancer cell line, the small molecule known as RITA (reactivation of p53 and induction of tumour cell apoptosis) that blocks the interaction between p53 and MDM2 was shown to restore the function of p53 and induce tumour cell apoptosis [151]. Indeed, there are several other small molecules that are used in therapies for tumours where p53 mutations have led to loss of the p53 DNA-binding function. For example, PRIMA (p53 reactivation and induction of massive apoptosis) 1 reactivates p53 and induces apoptosis [152]. Then, a small molecule with a similar structure to PRIMA-1 known as PRIMA^MET^ (APR-246) can also induce tumour cell apoptosis either alone or in combination with other chemotherapeutics. Furthermore, in multiple myeloma, MIRA (mutant p53 reactivation and induction of rapid apoptosis) 1 can restore the p53 function and p53-induced cancer cell apoptosis with a higher potency than PRIMA-1 [153]. Then, there is the small molecule RETRA (reactivation of transcriptional reporter activity) which not only restores the p53 function, but also increases the level of Tap73, a structural homologue of p53. However, the essential problem with these small molecules is the need for selective actions (in terms of restoration of the p53 function) that are directed only to the cancer cell.

## 7. Conclusions

Radiotherapy is the standard treatment of choice for patients with solid tumours and haematological malignancies although it induces the death not only of malignant cells, but also of healthy cells. However, cells can escape apoptotic death through activation of various survival factors. One of these factors is CREB, which is upregulated in various tumours treated with radiotherapy. This CREB upregulation leads to increased cell proliferation, reduced apoptosis, and enhanced cell migration and contributes to metastatic transformation of cells, as well as to angiogenesis. Along with CREB, ATF and NF-κB are also upregulated in different types of malignancies. Considering this, ionising radiation can also become a potential carcinogen, and thus it is important to consider the doses used. In addition, although the key role of CREB is evident in this context, it should not be considered the only therapeutic target. Indeed, further studies are needed to clarify other molecular players involved in mediating tumour radioresistance to identify novel, more efficient combined treatments designed on an individual patient and tumour basis.

## Figures and Tables

**Table 1:** Overexpression of the CREB/ATF family members and the related transcription factors: granulocyte–macrophage colony-stimulating factor (GM-CSF); activating protein 2 alfa (AP-2α); B-cell lymphoma 2 (Bcl-2); G-protein-coupled receptor 81 (GPR81); microRNA 1297 (mir-1297); P-glycoprotein (P-gp); G-protein-coupled receptor kinase 3 (GRK-3α). ↑ = increased expression; ↓ decreased expression.

↑ CREB/ATF	Cofactors	Type of Cancer
CREB	↑ GM-CSF	Acute myeloid leukaemia (AML)
CREB	↑ GM-CSF	Acute lymphoblastic leukaemia (ALL)
CREB/ATF-1	↓ AP-2α, ↑ Bcl-2	Human melanoma
CREB	↑ GPR-81	Breast cancer
CREB-1	↓ miR-1297	Gastric cancer
CREB	↑ Bcl-2, P-gp	Hepatocellular carcinoma (HCC)
CREB	↑ GRK-3α	Pancreatic cancer
CREB	↑ GRK-3α	Lung cancer
CREB	↑ GRK-3α	Neuroendocrine prostate cancer (NEPC)

## References

[1] El-Jaby S., Lewis B.J., Tomi L. (2020). A commentary on the impact of modelling results to inform mission planning and shield design. Life Sci. Sp. Res..

[2] Li M., Gonon G., Buonanno M., Autsavapromporn N., De Toledo S.M., Pain D., Azzam E.I. (2014). Health Risks of Space Exploration: Targeted and Nontargeted Oxidative Injury by High-Charge and High-Energy Particles. Antioxid. Redox Signal..

[3] Romero E., Francisco D. (2020). The NASA human system risk mitigation process for space exploration. Acta Astronaut..

[4] Sridharan D.M., Asaithamby A., Bailey S.M., Costes S.V., Doetsch P.W., Dynan W.S., Kronenberg A., Rithidech K.N., Saha J., Snijders A.M. (2015). Understanding cancer development processes after HZE-particle exposure: Roles of ROS, DNA damage repair and inflammation. Radiat. Res..

[5] Prasad B., Grimm D., Strauch S.M., Erzinger G.S., Corydon T.J., Lebert M., Magnusson N.E., Infanger M., Richter P., Krüger M. (2020). Influence of Microgravity on Apoptosis in Cells, Tissues, and Other Systems In Vivo and In Vitro. Int. J. Mol. Sci..

[6] Simonsen L.C., Slaba T.C., Guida P., Rusek A. (2020). NASA’s first ground-based Galactic Cosmic Ray Simulator: Enabling a new era in space radiobiology research. PLoS Biol..

[7] Barcellos-Hoff M.H., Blakely E.A., Burma S., Fornace A.J., Gerson S., Hlatky L., Kirsch D.G., Luderer U., Shay J., Wang Y. (2015). Concepts and challenges in cancer risk prediction for the space radiation environment. Life Sci. Sp. Res..

[8] Montminy M. (2003). Transcriptionl regulation by ciclic amp. Annu. Rev. Biochem..

[9] Papavassiliou A.G. (1994). The CREB/ATF family of transcription factors: Modulation by reversible phosphorylation. Anticancer Res..

[10] Niu M., Tabari E., Ni P., Su Z. (2018). Towards a map of cis-regulatory sequences in the human genome. Nucleic Acids Res..

[11] D’Auria F., Centurione L., Centurione M.A., Angelini A., Di Pietro R. (2017). Regulation of Cancer Cell Responsiveness to Ionizing Radiation Treatment by Cyclic AMP Response Element Binding Nuclear Transcription Factor. Front. Oncol..

[12] Lavrovsky Y., Schwartzman M.L., Levere R.D., Kappas A., Abraham N.G. (1994). Identification of binding sites for transcription factors NF-kappa B and AP-2 in the promoter region of the human heme oxygenase 1 gene. Proc. Natl. Acad. Sci. USA.

[13] Kogure Y., Kataoka K. (2017). Genetic alterations in adult T-cell leukemia/lymphoma. Cancer Sci..

[14] Cataldi A., Rapino M., Centurione L., Sabatini N., Grifone G., Garaci F., Rana R. (2003). NF-kB activation plays an antiapoptotic role in human leukemic K562 cells exposed to ionizing radiation. J. Cell. Biochem..

[15] Yu H., Aravindan N., Xu J., Natarajan M. (2017). Inter- and intra-cellular mechanism of NF-kB-dependent survival advantage and clonal expansion of radio-resistant cancer cells. Cell. Signal..

[16] Mortezaee K., Najafi M., Farhood B., Ahmadi A., Shabeeb D., Musa A.E. (2019). NF-κB targeting for overcoming tumor resistance and normal tissues toxicity. J. Cell. Physiol..

[17] Obrador E., Salvador R., Villaescusa J.I., Soriano J.M., Estrela J.M., Montoro A. (2020). Radioprotection and Radiomitigation: From the Bench to Clinical Practice. Biomedicines.

[18] Tang F.R., Loke W.K., Khoo B.C. (2017). Low-dose or low-dose-rate ionizing radiation–induced bioeffects in animal models. J. Radiat. Res..

[19] Spitz D.R., Azzam E.I., Jian Li J., Gius D. (2004). Metabolic oxidation/reduction reactions and cellular responses to ionizing radiation: A unifying concept in stress response biology. Cancer Metastasis Rev..

[20] Chaudhry M.A. (2006). Bystander effect: Biological endpoints and microarray analysis. Mutat. Res. Mol. Mech. Mutagen..

[21] Fleming A.M., Ding Y., Burrows C.J. (2017). Oxidative DNA damage is epigenetic by regulating gene transcription via base excision repair. Proc. Natl. Acad. Sci. USA.

[22] Tudek B., Winczura A., Janik J., Siomek A., Foksinski M., Oliński R. (2010). Involvement of oxidatively damaged DNA and repair in cancer development and aging. Am. J. Transl. Res..

[23] Valko M., Rhodes C.J., Moncol J., Izakovic M., Mazur M. (2006). Free radicals, metals and antioxidants in oxidative stress-induced cancer. Chem. Biol. Interact..

[24] Tubiana M. (2009). Can we reduce the incidence of second primary malignancies occurring after radiotherapy? A critical review. Radiother. Oncol..

[25] Bhat A.V., Hora S., Pal A., Jha S., Taneja R. (2018). Stressing the (Epi)Genome: Dealing with Reactive Oxygen Species in Cancer. Antioxid. Redox Signal..

[26] Bhutani N., Burns D.M., Blau H.M. (2011). DNA Demethylation Dynamics. Cell.

[27] Church M.C., Fleming A.B. (2018). A role for histone acetylation in regulating transcription elongation. Transcription.

[28] Zhang D., Tang Z., Huang H., Zhou G., Cui C., Weng Y., Liu W., Kim S., Lee S., Perez-Neut M. (2019). Metabolic regulation of gene expression by histone lactylation. Nature.

[29] Mladenov E., Li F., Zhang L., Klammer H., Iliakis G. (2018). Intercellular communication of DNA damage and oxidative status underpin bystander effects. Int. J. Radiat. Biol..

[30] Ilnytskyy Y., Kovalchuk O. (2011). Non-targeted radiation effects—An epigenetic connection. Mutat. Res. Mol. Mech. Mutagen..

[31] Albanese J., Dainiak N. (2000). Ionizing radiation alters Fas antigen ligand at the cell surface and on exfoliated plasma membrane-derived vesicles: Implications for apoptosis and intercellular signaling. Radiat. Res..

[32] Azzam E.I., de Toledo S.M., Gooding T., Little J.B. (1998). Intercellular communication is involved in the bystander regulation of gene expression in human cells exposed to very low fluences of alpha particles. Radiat. Res..

[33] Barcellos-Hoff M.H., Brooks A.L. (2001). Extracellular signaling through the microenvironment: A hypothesis relating carcinogenesis, bystander effects, and genomic instability. Radiat. Res..

[34] Nakamura N. (2020). A hypothesis: Radiation carcinogenesis may result from tissue injuries and subsequent recovery processes which can act as tumor promoters and lead to an earlier onset of cancer. Br. J. Radiol..

[35] Ishikawa K., Ishii H., Saito T. (2006). DNA Damage-Dependent Cell Cycle Checkpoints and Genomic Stability. DNA Cell Biol..

[36] Nakano H., Yonekawa H., Shinohara K. (2007). Threshold Level of p53 Required for the Induction of Apoptosis in X-Irradiated MOLT-4 Cells. Int. J. Radiat. Oncol..

[37] Heylmann D., Ponath V., Kindler T., Kaina B. (2021). Comparison of DNA repair and radiosensitivity of different blood cell populations. Sci. Rep..

[38] Portella L., Scala S. (2019). Ionizing radiation effects on the tumor microenvironment. Semin. Oncol..

[39] Matsumoto H., Hayashi S., Hatashita M., Ohnishi K., Shioura H., Ohtsubo T., Kitai R., Ohnishi T., Kano E. (2001). Induction of radioresistance by a nitric oxide-mediated bystander effect. Radiat. Res..

[40] González R., Molina-Ruiz F.J., Bárcena J.A., Padilla C.A., Muntané J. (2018). Regulation of Cell Survival, Apoptosis, and Epithelial-to-Mesenchymal Transition by Nitric Oxide-Dependent Post-Translational Modifications. Antioxid. Redox Signal..

[41] Coleman C.N., Eke I., Makinde A.Y., Chopra S., Demaria S., Formenti S.C., Martello S., Bylicky M., Mitchell J.B., Aryankalayil M.J. (2020). Radiation-induced Adaptive Response: New Potential for Cancer Treatment. Clin. Cancer Res..

[42] Chevalier F., Hamdi D.H., Saintigny Y., Lefaix J.-L. (2015). Proteomic overview and perspectives of the radiation-induced bystander effects. Mutat. Res. Mutat. Res..

[43] Suzuki K., Yamashita S. (2014). Radiation-Induced Bystander Response: Mechanism and Clinical Implications. Adv. Wound Care.

[44] Tazawa H., Kagawa S., Fujiwara T. (2016). p53 Replacement Therapy for Cancer. Recent Results Cancer Res..

[45] Acharya A., Rishi V., Moll J., Vinson C. (2006). Experimental identification of homodimerizing B-ZIP families in Homo sapiens. J. Struct. Biol..

[46] Zhang L., Yu H., Wang P., Ding Q., Wang Z. (2013). Screening of transcription factors with transcriptional initiation activity. Gene.

[47] Sapio L., Salzillo A., Ragone A., Illiano M., Spina A., Naviglio S. (2020). Targeting CREB in Cancer Therapy: A Key Candidate or One of Many? An Update. Cancers.

[48] Mayr B., Montminy M. (2001). Transcriptional regulation by the phosphorylation-dependent factor CREB. Nat. Rev. Mol. Cell Biol..

[49] Naqvi S., Martin K.J., Arthur J.S.C. (2014). CREB phosphorylation at Ser133 regulates transcription via distinct mechanisms downstream of cAMP and MAPK signalling. Biochem. J..

[50] Pregi N., Belluscio L.M., Berardino B.G., Castillo D.S., Cánepa E.T. (2017). Oxidative stress-induced CREB upregulation promotes DNA damage repair prior to neuronal cell death protection. Mol. Cell. Biochem..

[51] Kehat I., Hasin T., Aronheim A. (2006). The Role of Basic Leucine Zipper Protein-Mediated Transcription in Physiological and Pathological Myocardial Hypertrophy. Ann. N. Y. Acad. Sci..

[52] Medzhitov R., Horng T. (2009). Transcriptional control of the inflammatory response. Nat. Rev. Immunol..

[53] Cai D.H., Wang D., Keefer J., Yeamans C., Hensley K., Friedman A.D. (2008). C/EBPα:AP-1 leucine zipper heterodimers bind novel DNA elements, activate the PU.1 promoter and direct monocyte lineage commitment more potently than C/EBPα homodimers or AP-1. Oncogene.

[54] Hai T., Curran T. (1991). Cross-family dimerization of transcription factors Fos/Jun and ATF/CREB alters DNA binding specificity. Proc. Natl. Acad. Sci. USA.

[55] Wek R.C., Anthony T.G. (2006). EXtENDINg β cell survival by UPRegulating ATF4 translation. Cell Metab..

[56] Hai T., Hartman M.G. (2001). The molecular biology and nomenclature of the activating transcription factor/cAMP responsive element binding family of transcription factors: Activating transcription factor proteins and homeostasis. Gene.

[57] Gao S., Gao L., Wang S., Shi X., Yue C., Wei S., Zuo L., Zhang L., Qin X. (2021). ATF3 Suppresses Growth and Metastasis of Clear Cell Renal Cell Carcinoma by Deactivating EGFR/AKT/GSK3β/β-Catenin Signaling Pathway. Front. Cell Dev. Biol..

[58] Hasim M.S., Nessim C., Villeneuve P.J., Vanderhyden B.C., Dimitroulakos J. (2018). Activating Transcription Factor 3 as a Novel Regulator of Chemotherapy Response in Breast Cancer. Transl. Oncol..

[59] Smith S.G., Haynes K.A., Hegde A.N. (2020). Degradation of Transcriptional Repressor ATF4 during Long-Term Synaptic Plasticity. Int. J. Mol. Sci..

[60] Holmqvist P.-H., Mannervik M. (2013). Genomic occupancy of the transcriptional co-activators p300 and CBP. Transcription.

[61] Attar N., Kurdistani S.K. (2017). Exploitation of EP300 and CREBBP Lysine Acetyltransferases by Cancer. Cold Spring Harb. Perspect. Med..

[62] Mink S., Haenig B., Klempnauer K.H. (1997). Interaction and functional collaboration of p300 and C/EBPbeta. Mol. Cell. Biol..

[63] Burda P., Laslo P., Stopka T. (2010). The role of PU.1 and GATA-1 transcription factors during normal and leukemogenic hematopoiesis. Leukemia.

[64] Johannessen M., Delghandi M.P., Seternes O.M., Johansen B., Moens U. (2004). Synergistic activation of CREB-mediated transcription by forskolin and phorbol ester requires PKC and depends on the glutamine-rich Q2 transactivation domain. Cell. Signal..

[65] Hoesel B., Schmid J.A. (2013). The complexity of NF-κB signaling in inflammation and cancer. Mol. Cancer.

[66] Qin F., Fan Q., Yu P.K.N., Almahi W.A., Kong P., Yang M., Cao W., Nie L., Chen G., Han W. (2021). Properties and gene expression profiling of acquired radioresistance in mouse breast cancer cells. Ann. Transl. Med..

[67] Sokolova O., Naumann M. (2017). NF-κB Signaling in Gastric Cancer. Toxins.

[68] Vallabhapurapu S., Karin M. (2009). Regulation and Function of NF-κB Transcription Factors in the Immune System. Ann. Rev. Immunol..

[69] Shoji S., Hanada K., Takahashi M., Watanabe K., Yonemochi M., Tomabechi Y., Shirouzu M. (2020). The NF-κB regulator IκBβ exhibits different molecular interactivity and phosphorylation status from IκBα in an IKK2-catalysed reaction. FEBS Lett..

[70] Schmitz M.L., Bacher S., Kracht M. (2001). IκB-independent control of NF-κB activity by modulatory phosphorylations. Trends Biochem. Sci..

[71] Mukherjee S.P., Behar M., Birnbaum H.A., Hoffmann A., Wright P.E., Ghosh G. (2013). Analysis of the RelA:CBP/p300 Interaction Reveals Its Involvement in NF-κB-Driven Transcription. PLoS Biol..

[72] Ono H., Basson M.D., Ito H. (2016). P300 inhibition enhances gemcitabine-induced apoptosis of pancreatic cancer. Oncotarget.

[73] Dyson H.J., Wright P.E. (2016). Role of Intrinsic Protein Disorder in the Function and Interactions of the Transcriptional Coactivators CREB-binding Protein (CBP) and p300. J. Biol. Chem..

[74] Huante-Mendoza A., Silva-García O., Oviedo-Boyso J., Hancock R.E.W., Baizabal-Aguirre V.M. (2016). Peptide IDR-1002 Inhibits NF-κB Nuclear Translocation by Inhibition of IκBα Degradation and Activates p38/ERK1/2–MSK1-Dependent CREB Phosphorylation in Macrophages Stimulated with Lipopolysaccharide. Front. Immunol..

[75] Wen A.Y., Sakamoto K.M., Miller L.S. (2010). The Role of the Transcription Factor CREB in Immune Function. J. Immunol..

[76] Grossman S.R. (2001). p300/CBP/p53 interaction and regulation of the p53 response. Eur. J. Biochem..

[77] Liu H., Deng X., Shyu Y.J., Li J.J., Taparowsky E.J., Hu C.-D. (2006). Mutual regulation of c-Jun and ATF2 by transcriptional activation and subcellular localization. EMBO J..

[78] Basu S., Murphy M.E. (2016). Genetic Modifiers of the p53 Pathway. Cold Spring Harb. Perspect. Med..

[79] Vaseva A.V., Moll U.M. (2009). The mitochondrial p53 pathway. Biochim. Biophys. Acta-Bioenerg..

[80] Reinhardt H.C., Schumacher B. (2012). The p53 network: Cellular and systemic DNA damage responses in aging and cancer. Trends Genet..

[81] Pflaum J., Schlosser S., Müller M. (2014). p53 Family and Cellular Stress Responses in Cancer. Front. Oncol..

[82] Lavin M.F., Gueven N. (2006). The complexity of p53 stabilization and activation. Cell Death Differ..

[83] Joruiz S.M., Bourdon J.-C. (2016). p53 Isoforms: Key Regulators of the Cell Fate Decision. Cold Spring Harb. Perspect. Med..

[84] Levine A.J., Oren M. (2009). The first 30 years of p53: Growing ever more complex. Nat. Rev. Cancer.

[85] Chasov V., Mirgayazova R., Zmievskaya E., Khadiullina R., Valiullina A., Stephenson Clarke J., Rizvanov A., Baud M.G.J., Bulatov E. (2020). Key Players in the Mutant p53 Team: Small Molecules, Gene Editing, Immunotherapy. Front. Oncol..

[86] Mantovani F., Collavin L., Del Sal G. (2019). Mutant p53 as a guardian of the cancer cell. Cell Death Differ..

[87] Mogensen T.H. (2019). IRF and STAT Transcription Factors—From Basic Biology to Roles in Infection, Protective Immunity, and Primary Immunodeficiencies. Front. Immunol..

[88] Steven A., Friedrich M., Jank P., Heimer N., Budczies J., Denkert C., Seliger B. (2020). What turns CREB on? And off? And why does it matter?. Cell. Mol. Life Sci..

[89] Siu Y.-T., Jin D.-Y. (2007). CREB—A real culprit in oncogenesis. FEBS J..

[90] D’Auria F., Di Pietro R. (2013). Role of CREB Protein Family Members in Human Haematological Malignancies. Cancer Treatment—Conventional and Innovative Approaches.

[91] Cho E.-C., Mitton B., Sakamoto K. (2011). CREB and Leukemogenesis. Crit. Rev. Oncog..

[92] Cataldi A., Di Giacomo V., Rapino M., Genovesi D., Rana R.A. (2006). Cyclic Nucleotide Response Element Binding Protein (CREB) Activation Promotes Survival Signal in Human K562 Erythroleukemia Cells Exposed to Ionising Radiation/Etoposide Combined Treatment. J. Radiat. Res..

[93] Di Nisio C., Sancilio S., Di Giacomo V., Rapino M., Sancillo L., Genovesi D., Di Siena A., Rana R.A., Cataldi A., Di Pietro R. (2016). Involvement of cyclic-nucleotide response element-binding family members in the radiation response of Ramos B lymphoma cells. Int. J. Oncol..

[94] Steven A., Seliger B. (2016). Control of CREB expression in tumors: From molecular mechanisms and signal transduction pathways to therapeutic target. Oncotarget.

[95] Jean D., Menashe B.-E., Bar-Eli M. (2000). Regulation of tumor growth and metastasis of human melanoma by the CREB transcription factor family. Mol. Cell. Biochem..

[96] Sarkar D., Leung E.Y., Baguley B.C., Finlay G.J., Askarian-Amiri M.E. (2015). Epigenetic regulation in human melanoma: Past and future. Epigenetics.

[97] Braeuer R.R., Zigler M., Villares G.J., Dobroff A.S., Bar-Eli M. (2011). Transcriptional control of melanoma metastasis: The importance of the tumor microenvironment. Semin. Cancer Biol..

[98] Lee Y.J., Shin K.J., Park S.-A., Park K.S., Park S., Heo K., Seo Y.-K., Noh D.-Y., Ryu S.H., Suh P.-G. (2016). G-protein-coupled receptor 81 promotes a malignant phenotype in breast cancer through angiogenic factor secretion. Oncotarget.

[99] Caravatta L., Sancilio S., di Giacomo V., Rana R., Cataldi A., Di Pietro R. (2008). PI3-K/Akt-dependent activation of cAMP-response element-binding (CREB) protein in Jurkat T leukemia cells treated with TRAIL. J. Cell. Physiol..

[100] Shankar D.B., Cheng J.C., Kinjo K., Federman N., Moore T.B., Gill A., Rao N.P., Landaw E.M., Sakamoto K.M. (2005). The role of CREB as a proto-oncogene in hematopoiesis and in acute myeloid leukemia. Cancer Cell.

[101] Wang Y.W., Chen X., Gao J.W., Zhang H., Ma R.R., Gao Z.H., Gao P. (2015). High expression of cAMP responsive element binding protein 1 (CREB1) is associated with metastasis, tumor stage and poor outcome in gastric cancer. Oncotarget.

[102] Abramovitch R., Tavor E., Jacob-Hirsch J., Zeira E., Amariglio N., Pappo O., Rechavi G., Galun E., Honigman A. (2004). A Pivotal Role of Cyclic AMP-Responsive Element Binding Protein in Tumor Progression. Cancer Res..

[103] Ye C.-G., Yeung J.H.-K., Huang G.-L., Cui P., Wang J., Zou Y., Zhang X.-N., He Z.-W., Cho C.-H. (2013). Increased glutathione and mitogen-activated protein kinase phosphorylation are involved in the induction of doxorubicin resistance in hepatocellular carcinoma cells. Hepatol. Res..

[104] Belkhiri A., Dar A.A., Zaika A., Kelley M., El-Rifai W. (2008). t-Darpp Promotes Cancer Cell Survival by Up-regulation of Bcl2 through Akt-Dependent Mechanism. Cancer Res..

[105] Sang M., Hulsurkar M., Zhang X., Song H., Zheng D., Zhang Y., Li M., Xu J., Zhang S., Ittmann M. (2016). GRK3 is a direct target of CREB activation and regulates neuroendocrine differentiation of prostate cancer cells. Oncotarget.

[106] Darrington R.S., Campa V.M., Walker M.M., Bengoa-Vergniory N., Gorrono-Etxebarria I., Uysal-Onganer P., Kawano Y., Waxman J., Kypta R.M. (2012). Distinct expression and activity of GSK-3α and GSK-3β in prostate cancer. Int. J. Cancer.

[107] Banerji V., Frumm S.M., Ross K.N., Li L.S., Schinzel A.C., Hahn C.K., Kakoza R.M., Chow K.T., Ross L., Alexe G. (2012). The intersection of genetic and chemical genomic screens identifies GSK-3α as a target in human acute myeloid leukemia. J. Clin. Investig..

[108] Bang D., Wilson W., Ryan M., Yeh J.J., Baldwin A.S. (2013). GSK-3α Promotes Oncogenic KRAS Function in Pancreatic Cancer via TAK1–TAB Stabilization and Regulation of Noncanonical NF-κB. Cancer Discov..

[109] Park S.-A., Lee J.W., Herbst R.S., Koo J.S. (2016). GSK-3α Is a Novel Target of CREB and CREB-GSK-3α Signaling Participates in Cell Viability in Lung Cancer. PLoS ONE.

[110] Cataldi A., Di Giacomo V., Rapino M., Zara S., Rana R.A. (2009). Ionizing Radiation Induces Apoptotic Signal Through Protein Kinase Cδ (delta) and Survival Signal Through Akt and Cyclic-Nucleotide Response Element-Binding Protein (CREB) in Jurkat T Cells. Biol. Bull..

[111] Katanyutanon S., Wu R., Wang P. (2008). The effect of whole-body radiation on blood levels of gastrointestinal peptides in the rat. Int. J. Clin. Exp. Med..

[112] Chen X., Shen B., Xia L., Khaletzkiy A., Wong J.Y.C., Li J.J., Chu D. (2002). Activation of nuclear factor κB in radioresistance of TP53-inactive human keratinocytes. Cancer Res..

[113] Chua J.S., Liew H.P., Guo L., Lane D.P. (2015). Tumor-specific signaling to p53 is mimicked by Mdm2 inactivation in zebrafish: Insights from mdm2 and mdm4 mutant zebrafish. Oncogene.

[114] Stewart-Ornstein J., Iwamoto Y., Miller M.A., Prytyskach M.A., Ferretti S., Holzer P., Kallen J., Furet P., Jambhekar A., Forrester W.C. (2021). p53 dynamics vary between tissues and are linked with radiation sensitivity. Nat. Commun..

[115] Qi J.-P., Shao S.-H., Li D.-D., Zhou G.-P. (2007). A dynamic model for the p53 stress response networks under ion radiation. Amino Acids.

[116] Qi J., Shao S., Shen Y. (2008). Cellular responding DNA damage: An improved modeling of P53 gene regulatory networks under ion radiation (IR). Appl. Math. Comput..

[117] Macaluso M., Montanari M., Cinti C., Giordano A. (2005). Modulation of Cell Cycle Components by Epigenetic and Genetic Events. Semin. Oncol..

[118] Teufel D.P., Bycroft M., Fersht A.R. (2009). Regulation by phosphorylation of the relative affinities of the N-terminal transactivation domains of p53 for p300 domains and Mdm2. Oncogene.

[119] Sokolov M., Neumann R. (2018). Changes in gene expression as one of the key mechanisms involved in radiation-induced bystander effect (Review). Biomed. Rep..

[120] DeVries-Seimon T.A., Ohm A.M., Humphries M.J., Reyland M.E. (2007). Induction of Apoptosis Is Driven by Nuclear Retention of Protein Kinase Cδ. J. Biol. Chem..

[121] Bluwstein A., Kumar N., Léger K., Traenkle J., van Oostrum J., Rehrauer H., Baudis M., Hottiger M.O. (2013). PKC signaling prevents irradiation-induced apoptosis of primary human fibroblasts. Cell Death Dis..

[122] Martelli A.M., Evangelisti C., Nyakern M., Manzoli F.A. (2006). Nuclear protein kinase C. Biochim. Biophys. Acta.

[123] Xue Y., Ren J., Gao X., Jin C., Wen L., Yao X. (2008). GPS 2.0, a tool to predict kinase-specific phosphorylation sites in hierarchy. Mol. Cell. Proteomics.

[124] Nickoloff J.A., Sharma N., Taylor L. (2020). Clustered DNA Double-Strand Breaks: Biological Effects and Relevance to Cancer Radiotherapy. Genes.

[125] Alam M., Kashyap T., Pramanik K.K., Singh A.K., Nagini S., Mishra R. (2017). The elevated activation of NFκB and AP-1 is correlated with differential regulation of Bcl-2 and associated with oral squamous cell carcinoma progression and resistance. Clin. Oral Investig..

[126] Voboril R., Rychterova V., Voborilova J., Kubecova M., Fanta J., Dvorak J. (2016). NF-κB/p65 expression before and after treatment in rectal cancer patients undergoing neoadjuvant (chemo)radiotherapy and surgery: Prognostic marker for disease progression and survival. Neoplasma.

[127] Basu S., Rosenzweig K.R., Youmell M., Price B.D. (1998). The DNA-Dependent Protein Kinase Participates in the Activation of NFκB Following DNA Damage. Biochem. Biophys. Res. Commun..

[128] Madhusoodhanan R., Natarajan M.N., Veeraraghavan J., Herman T.S., Aravindan N. (2009). NFκB activity and transcriptional responses in human breast adenocarcinoma cells after single and fractionated irradiation. Cancer Biol. Ther..

[129] Ramos P.M.M., Pezuk J.A., Castro-Gamero A.M., Oliveira H.F., Scrideli C.A., Umezawa K., Tone L.G. (2018). Antineoplastic Effects of NF-κB Inhibition by DHMEQ (Dehydroxymethylepoxyquinomicin) Alone and in Co-treatment with Radio-and Chemotherapy in Medulloblastoma Cell Lines. Anticancer. Agents Med. Chem..

[130] Samuel T., Fadlalla K., Gales D.N., Putcha B.D., Manne U. (2014). Variable NF-κB pathway responses in colon cancer cells treated with chemotherapeutic drugs. BMC Cancer.

[131] Chen M., Singh A.K., Repasky E.A. (2020). Highlighting the Potential for Chronic Stress to Minimize Therapeutic Responses to Radiotherapy through Increased Immunosuppression and Radiation Resistance. Cancers.

[132] Nelson E.C., Cambio A.J., Yang J.C., Ok J.-H., Lara P.N., Evans C.P. (2007). Clinical implications of neuroendocrine differentiation in prostate cancer. Prostate Cancer Prostatic.

[133] Huang Y.-H., Zhang Y.-Q., Huang J.-T. (2019). Neuroendocrine cells of prostate cancer: Biologic functions and molecular mechanisms. Asian J. Androl..

[134] Liu M., Guyot-Sionnest P. (2005). Mechanism of silver(I)-assisted growth of gold nanorods and bipyramids. J. Phys. Chem. B.

[135] Deng X., Liu H., Huang J., Cheng L., Keller E.T., Parsons S.J., Hu C.-D. (2008). Ionizing Radiation Induces Prostate Cancer Neuroendocrine Differentiation through Interplay of CREB and ATF2: Implications for Disease Progression. Cancer Res..

[136] Di Pietro R., Fang H., Fields K., Miller S., Flora M., Petricoin E.C., Dveksler G., Rana R.A., Grimley P.M. (2006). Peroxiredoxin Genes are Not Induced in Myeloid Leukemia Cells Exposed to Ionizing Radiation. Int. J. Immunopathol. Pharmacol..

[137] Rhee S.G. (2016). Overview on Peroxiredoxin. Mol. Cells.

[138] Wang T., Diaz A.J.G., Yen Y. (2014). The role of peroxiredoxin II in chemoresistance of breast cancer cells. Breast Cancer Targ. Ther..

[139] Alper O., Bergmann-Leitner E.S., Abrams S., Cho-Chung Y.S. (2001). Apoptosis, growth arrest and suppression of invasiveness by CRE-decoy oligonucleotide in ovarian cancer cells: Protein kinase A downregulation and cytoplasmic export of CRE-binding proteins. Mol. Cell. Biochem..

[140] Steven A., Leisz S., Massa C., Iezzi M., Lattanzio R., Lamolinara A., Bukur J., Müller A., Hiebl B., Holzhausen H.-J. (2013). HER-2/neu Mediates Oncogenic Transformation via Altered CREB Expression and Function. Mol. Cancer Res..

[141] Xie F., Li B.X., Kassenbrock A., Xue C., Wang X., Qian D.Z., Sears R.C., Xiao X. (2015). Identification of a Potent Inhibitor of CREB-Mediated Gene Transcription with Efficacious in Vivo Anticancer Activity. J. Med. Chem..

[142] Tsolou A., Liousia M., Kalamida D., Pouliliou S., Giatromanolaki A., Koukourakis M. (2017). Inhibition of IKK-NFκB pathway sensitizes lung cancer cell lines to radiation. Cancer Biol. Med..

[143] Molavi Pordanjani S., Jalal Hosseinimehr S. (2016). The Role of NF-κB Inhibitors in Cell Response to Radiation. Curr. Med. Chem..

[144] Hara K., Takeda A., Tsurugai Y., Saigusa Y., Sanuki N., Eriguchi T., Maeda S., Tanaka K., Numata K. (2019). Radiotherapy for Hepatocellular Carcinoma Results in Comparable Survival to Radiofrequency Ablation: A Propensity Score Analysis. Hepatology.

[145] Fuchs-Tarlovsky V. (2013). Role of antioxidants in cancer therapy. Nutrition.

[146] Impicciatore G., Sancilio S., Miscia S., Di Pietro R. (2010). Nutlins and Ionizing Radiation in Cancer Therapy. Curr. Pharm. Des..

[147] Nayak S.K., Khatik G.L., Narang R., Monga V., Chopra H.K. (2017). p53-Mdm2 Interaction Inhibitors as Novel Nongenotoxic Anticancer Agents. Curr. Cancer Drug Targ..

[148] Zauli G., Celeghini C., Melloni E., Voltan R., Ongari M., Tiribelli M., di Iasio M.G., Lanza F., Secchiero P. (2012). The sorafenib plus nutlin-3 combination promotes synergistic cytotoxicity in acute myeloid leukemic cells irrespectively of FLT3 and p53 status. Haematologica.

[149] Gu L., Zhu N., Findley H.W., Zhou M. (2008). MDM2 antagonist nutlin-3 is a potent inducer of apoptosis in pediatric acute lymphoblastic leukemia cells with wild-type p53 and overexpression of MDM2. Leukemia.

[150] Zauli G., Voltan R., Bosco R., Melloni E., Marmiroli S., Rigolin G.M., Cuneo A., Secchiero P. (2011). Dasatinib Plus Nutlin-3 Shows Synergistic Antileukemic Activity in Both p53 wild-type and p53 mutated B Chronic Lymphocytic Leukemias by Inhibiting the Akt Pathway. Clin. Cancer Res..

[151] Roh J.L., Ko J.H., Moon S.J., Ryu C.H., Choi J.Y., Koch W.M. (2012). The p53-reactivating small-molecule RITA enhances cisplatin-induced cytotoxicity and apoptosis in head and neck cancer. Cancer Lett..

[152] Wiman K.G. (2010). Pharmacological reactivation of mutant p53: From protein structure to the cancer patient. Oncogene.

[153] Hientz K., Mohr A., Bhakta-Guha D., Efferth T. (2017). The role of p53 in cancer drug resistance and targeted chemotherapy. Oncotarget.

